# Antibacterial and Cellular Behaviors of Novel Zinc-Doped Hydroxyapatite/Graphene Nanocomposite for Bone Tissue Engineering

**DOI:** 10.3390/ijms22179564

**Published:** 2021-09-03

**Authors:** H. Maleki-Ghaleh, M. H. Siadati, A. Fallah, B. Koc, M. Kavanlouei, P. Khademi-Azandehi, E. Moradpur-Tari, Y. Omidi, J. Barar, Y. Beygi-Khosrowshahi, Alan P. Kumar, K. Adibkia

**Affiliations:** 1 Research Center for Pharmaceutical Nanotechnology, Biomedicine Institute, Tabriz University of Medical Sciences, Tabriz 51664-14766, Iran; h_maleki@sut.ac.ir (H.M.-G.); jbarar@tbzmed.ac.ir (J.B.); 2 Faculty of Materials Science and Engineering, K. N. Toosi University of Technology, Tehran 19919-43344, Iran; siadati@kntu.ac.ir; 3 Faculty of Engineering and Natural Sciences, Sabanci University, Istanbul 34956, Turkey; ali.fallah@sabanciuniv.edu (A.F.); bahattin.koc@sabanciuniv.edu (B.K.); 4 Nanotechnology Research and Application Center (SUNUM), Sabanci University, Istanbul 34956, Turkey; 5 Materials Engineering Department, Faculty of Engineering, Urmia University, Urmia 57561-51818, Iran; m.kavanlouei@urmia.ac.ir; 6 Research Center for Advanced Materials, Faculty of Materials Engineering, Sahand University of Technology, Tabriz 51335-1996, Iran; p_khademi@hotmail.com; 7 Materials Engineering Department, Faculty of Engineering, Tarbiat Modares University, Tehran 14115-111, Iran; e.moradpour@modares.ac.ir; 8 Department of Pharmaceutical Sciences, College of Pharmacy, Nova Southeastern University, Fort Lauderdale, FL 33314, USA; yomidi@nova.edu; 9 Department of Pharmaceutics, Faculty of Pharmacy, Tabriz University of Medical Sciences, Tabriz 51664-14766, Iran; 10 Department of Chemical Engineering, Faculty of Engineering, Azarbaijan Shahid Madani University, Tabriz 53751-71379, Iran; yonesbeygi@gmail.com; 11 Department of Pharmacology, Yong Loo Lin School of Medicine, National University of Singapore, Singapore 117599, Singapore; 12 NUS Centre for Cancer Research, Yong Loo Lin School of Medicine, National University of Singapore, Singapore 117597, Singapore

**Keywords:** antibacterial, biocompatibility, nanocomposite, hydroxyapatite, zinc, graphene

## Abstract

Bacteria are one of the significant causes of infection in the body after scaffold implantation. Effective use of nanotechnology to overcome this problem is an exciting and practical solution. Nanoparticles can cause bacterial degradation by the electrostatic interaction with receptors and cell walls. Simultaneously, the incorporation of antibacterial materials such as zinc and graphene in nanoparticles can further enhance bacterial degradation. In the present study, zinc-doped hydroxyapatite/graphene was synthesized and characterized as a nanocomposite material possessing both antibacterial and bioactive properties for bone tissue engineering. After synthesizing the zinc-doped hydroxyapatite nanoparticles using a mechanochemical process, they were composited with reduced graphene oxide. The nanoparticles and nanocomposite samples were extensively investigated by transmission electron microscopy, X-ray diffraction, and Raman spectroscopy. Their antibacterial behaviors against Escherichia coli and Staphylococcus aureus were studied. The antibacterial properties of hydroxyapatite nanoparticles were found to be improved more than 2.7 and 3.4 times after zinc doping and further compositing with graphene, respectively. In vitro cell assessment was investigated by a cell viability test and alkaline phosphatase activity using mesenchymal stem cells, and the results showed that hydroxyapatite nanoparticles in the culture medium, in addition to non-toxicity, led to enhanced proliferation of bone marrow stem cells. Furthermore, zinc doping in combination with graphene significantly increased alkaline phosphatase activity and proliferation of mesenchymal stem cells. The antibacterial activity along with cell biocompatibility/bioactivity of zinc-doped hydroxyapatite/graphene nanocomposite are the highly desirable and suitable biological properties for bone tissue engineering successfully achieved in this work.

## 1. Introduction

Bacterial infections caused by orthopedic surgeries (such as implantation of bone scaffold and artificial joints) can lead to allergies, inflammation, and necrosis of tissues in the implanted area [1,2]. Damage in tissues could even require re-surgeries to remove the implant or scaffolds [3,4]. Using innovative materials to construct implants or bone scaffolds possessing antibacterial properties together with biocompatibility is an attractive solution to control bacterial infections [4,5]. Considering the biomaterials used for bone implants and scaffolds, hydroxyapatite (HA) is one of the preferred choices [6,7]. Possessing chemical composition of Ca_10_(PO_4_)_6_(OH)_2_, HA is one of the main components of bone (nearly 70%) which forms the mineral part of bones tissues [8]. Because HA is biocompatible, non-toxic, and non-inflammatory at the same time, it is one of the most favored biomaterials for fabricating bone scaffolds [9,10]. Due to its osteoinductivity and osteoconductivity properties, HA in scaffolds can accelerate new bone tissue formation [11]. As a significant factor in advancing the medical engineering field, nanotechnology can create superior capabilities in biomaterials by modifying their structures [12,13]. For example, upon modifying the crystal structure of HA by doping with antibacterial elements, and further compositing it with carbon nanomaterials, can not only improve the new HA nanocomposite material’s bioactivity but also its other biological features such as antibacterial properties [14,15]. Zinc (Zn) has a unique state among the elements that have antibacterial properties [16]. Zn causes bacterial death due to mechanisms such as reactive oxygen species, such as peroxides and hydroxides, and direct reactions with bacterial proteins such as phospholipids [16,17]. Therefore, Zn as a doping element promotes HA nanoparticles’ antibacterial potency while increasing its bioactivity [17]. Recent studies showed that Zn can boost osteoblast cell proliferation and osteogenic differentiation [18,19]. During the natural bone remodeling process, Zn is released from the bone. Research has shown that Zn released from bone reduces osteoclast resorption and increases osteoblast ossification [20]. Zn also accelerates the hydrolysis of phosphomonoesters to mineral phosphates, creating a suitable alkaline environment for mineral phosphate mineralization on the extracellular matrix (ECM) [21]. Moreover, Zn affects mitochondrial anaplerotic reactions by decreasing lactate, and increasing intermediates of the Krebs cycle; these increase citrate accumulation and deposition of bone apatite. As a result, due to increased citrate anabolism, mineralization occurs more rapidly [22]. The bioenergetic changes of mitochondria and transportation of calcium and citrate in the mitochondria and cytoplasm are manifest during stem cell differentiation [23]. Meanwhile, genes related to mitochondrial biogenesis, such as ERRα and PGC-1α, are activated to increase anaplerotic reactions to intermediate replenishment of the Krebs cycle [24]. As a result, it causes compounds such as α-ketoglutarate to have high concentrations during osteogenic differentiation. In addition, glutaminolysis-related genes such as GLAST, GLS, and GDH are also activated to produce more α-ketoglutarate. More expression of CS and CTP genes during differentiation helps increase citrate anabolism and citrate transportation, leading to citrate catabolism reduction [22]. The cooperation of Zn with Runx2 and OSX results in the Zn-Runx2/OSX-ZIP1 regulatory axis formation, increasing Zn transporter protein expression (ZIP1). An increase in ZIP1 leads to an influx of Zn into the cell, enhancing the proliferation and differentiation signaling pathways such as TGF-β and BMP. Since extracellular citrate deposition is derived from the metabolism of the mitochondrial Krebs cycle [25], induction of ZIP1 can reduce citrate catabolism by suppressing mitochondrial aconitase activity. Citrate catabolism reduction results in enhanced citrate accumulation and deposition, a crucial factor in bone-strengthening [26].

Meanwhile, graphene-based nanomaterials, due to their unique physical and chemical properties, have exceptional potential for use in biomedicine, such as drug delivery, cancer therapy, and tissue engineering [27,28]. For example, in bone tissue engineering, reduced graphene oxide (rGO) provides significant biological performance, such as improving osteoblastic differentiation and antibacterial activity [29,30]. Recent research work proved that graphene nanomaterials can not only deactivate bacteria with impressive results, but at the same time it has shown high osteoconductivity and osteoinductivity capacities for regulating osteogenic differentiation [31,32]. Moreover, graphene supports bone cell growth and osteogenic differentiation due to its high stiffness modulus and is suitable for bone scaffolds [33]. By integration of graphene and Zn-doped HA in the form of a nanocomposite, the synergistic effect of nanomaterial can be expected simultaneously to show two behaviors: antibacterial and bioactivity. In this study, Zn-doped HA nanoparticles were synthesized using a mechanochemical process, and rGO was composited to them by photochemical reduction to form a novel HA-based nanocomposite. The nanoparticles were characterized by transmission electron microscopy (TEM), X-ray diffraction (XRD), and Raman spectroscopy, and evaluation of their cellular behavior was conducted using mesenchymal stem cells (MSCs) by cell viability tests and alkaline phosphatase (ALP) activity. The antibacterial behaviors of nanoparticles were also studied with Escherichia coli (*E. coli*) and Staphylococcus aureus (*S. aureus*).

## 2. Results

### 2.1. Characterization of Nanoparticles

**Figure 1:**
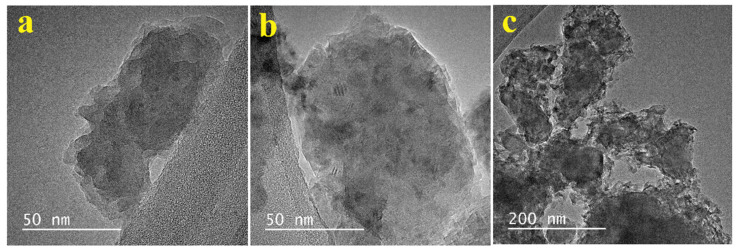
TEM images of (**a**) HA, and (**b**) ZnHA nanoparticles showing flake-like morphology, and (**c**) ZnHA-rGO showing ZnHA nanoparticles composited with rGO sheets.

**Figure 2:**
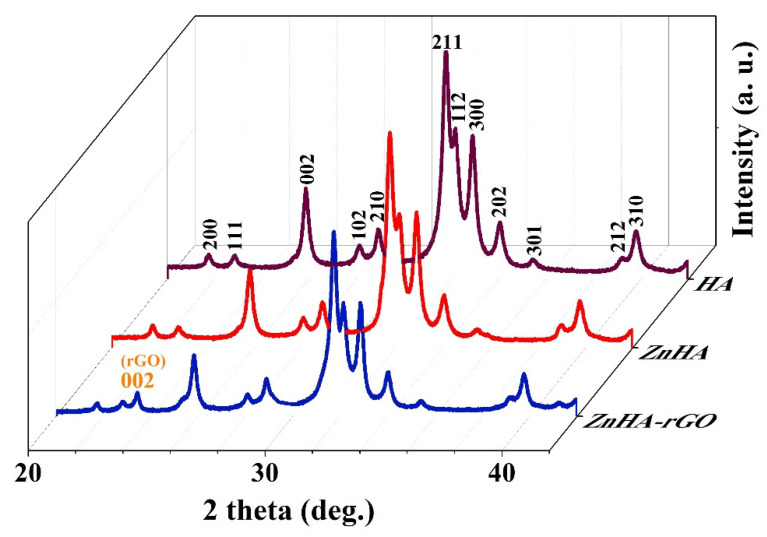
XRD patterns of HA, ZnHA, and ZnHA-rGO nanoparticles. The diffraction pattern of HA nanoparticles was well in accordance with the standard pattern of HA (JCPDS card No. 09–0432); hexagonal structure. The XRD patterns of ZnHA and ZnHA-rGO nanoparticles were highly similar to that of HA nanoparticles. A peak corresponding to graphene nanosheets (002) was seen in ZnHA-rGO.

The X-ray photoelectron spectroscopy (XPS), XRD, and attenuated total reflectance-Fourier transform infrared (ATR-FTIR) characterizations of the nanoparticles have been thoroughly studied in the previous work [34]. The TEM images for (a) HA, (b) ZnHA, and (c) ZnHA-rGO nanoparticles are presented in Figure 1. As shown in Figure 1a,b, the HA and ZnHA nanoparticles have a flake-like or quasi-spherical shape with a rough surface. Figure 1c demonstrates the ZnHA-rGO sample; the ZnHA nanoparticles formed a nanocomposite with the rGO sheets. Furthermore, the phase analysis results (XRD patterns) in Figure 2 show the hexagonal structure for HA nanoparticles (JCPDS 09-432). The ZnHA nanoparticles also show a hexagonal structure like HA [35,36,37,38]. At the same time, compositing the ZnHA nanoparticles with rGO nanosheets did not affect the crystal structure, but a new peak associated with graphene nanosheets (002) appeared [39].

**Figure 3:**
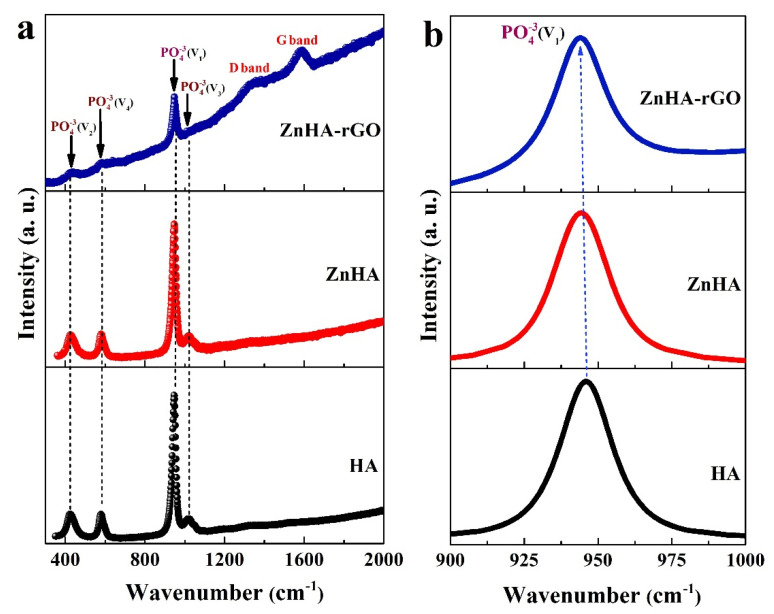
(**a**) Raman spectra of HA, ZnHA, and ZnHA-rGO nanoparticles, (**b**) Raman spectra in 900–1000 cm^−1^ range. Raman spectra indicate that the rGO nanosheets wrap the surface of ZnHA nanoparticles. Zn doping in the HA structure and its compositing with rGO has made PO_4_^3−^ bands lower and broader.

Figure 3a shows the Raman spectra for HA, ZnHA, and ZnHA-rGO nanoparticles. Representative bands of the HA sample are associated with the doubly degenerate bending mode v_2_(PO_4_^3−^) (426 cm^−1^), triply degenerate bending mode v_4_(PO_4_^3−^) (583 cm^−1^), symmetric stretching mode v_1_(PO_4_^3−^) (951 cm^−1^), and asymmetric stretching mode v_3_(PO_4_^3−^) (1024 cm^−1^) [40,41,42]. These bands are also well-observed in the ZnHA and ZnHA-rGO spectra, indicating the stability of the molecular structure of HA after doping and compositing with Zn and rGO, respectively. In the ZnHA-rGO sample, two broad bands are related with the vibration of carbon atoms in the graphene lattice, the D and G bands in the range of 1347 and 1601 cm^−1^, respectively [43]. The D band is associated with lattice distortions, and the G band is related to sp^2^ hybridized carbon–carbon bonds in graphene [43]. Furthermore, Zn doping in the HA structure and its compositing with rGO has made PO_4_^3−^ bands lower and broader. This is well seen in Figure 3b for the v_1_(PO_4_^3−^) band in the region of 950 cm^−1^. The difference in ionic radius between Zn^2+^ and Ca^2+^ can distort the PO_4_^3−^ structure [40,41]. Moreover, the interaction between the rGO atoms with the surface atoms of HA nanoparticles can affect the structure of PO_4_^3−^ [34].

### 2.2. In-Vitro Cell Analysis

**Figure 4:**
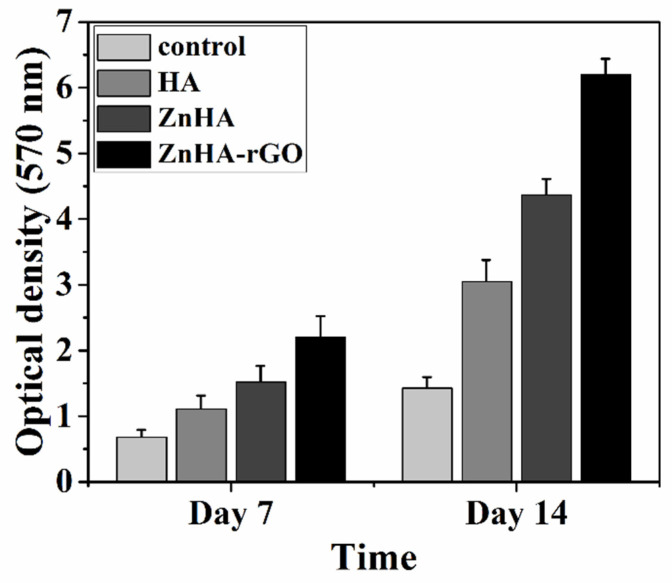
The viability of MSCs incubated in culture media treated by the HA, ZnHA, and ZnHA-rGO nanoparticles using MTT assay. During the incubation period (14 days), the presence of nanoparticles caused an appreciable increase in cell proliferation, especially ZnHA-rGO, compared to the non-treated control.

The effect of nanoparticles on MSC proliferation was investigated for 7 and 14 days by MTT (3-(4,5-dimethylthiazol-2-yl)-2,5-diphenyltetrazolium bromide, a tetrazole) assay. As shown in Figure 4, the MSC proliferation in culture media treated by the nanoparticles is higher than in the control one. Moreover, doping HA nanoparticles with Zn increased MSC proliferation, and it was further enhanced by the addition of rGO.

**Figure 5:**
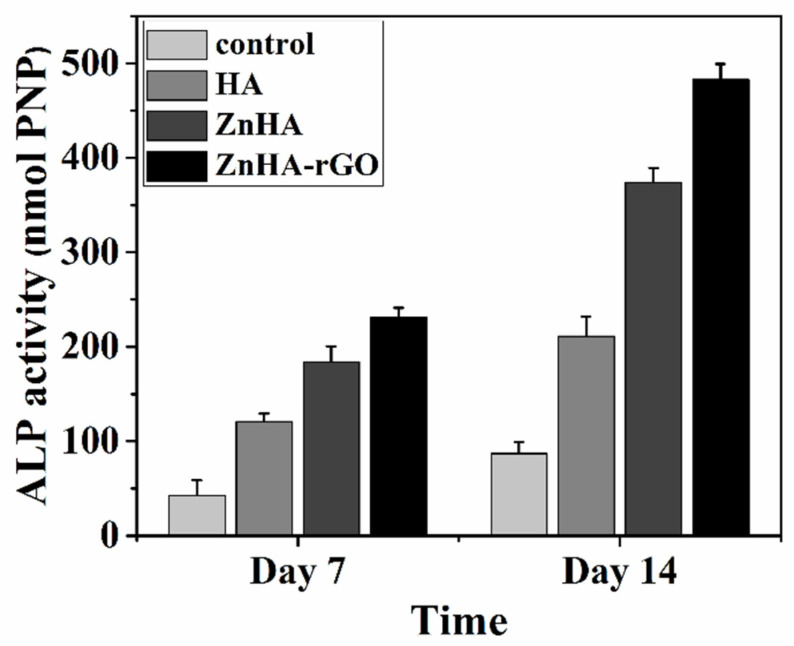
ALP activity of MSCs in culture media treated by the HA, ZnHA, and ZnHA-rGO nanoparticles. Incubation with ZnHA-rGO nanoparticles for 7 to 14 days significantly induced ALP activity.

ALP plays a vital function in the matrix mineralization process during bone formation. The bone differentiation capacity of MSCs was assessed by ALP activity after 7 and 14 days of incubation. The ALP activity in culture media treated with HA, ZnHA, and ZnHA-rGO nanoparticles compared with the control media is presented in Figure 5. The ALP activity of all three nanoparticles increased with incubation time, considerably higher for ZnHA-rGO.

### 2.3. Antibacterial Assay

**Figure 6:**
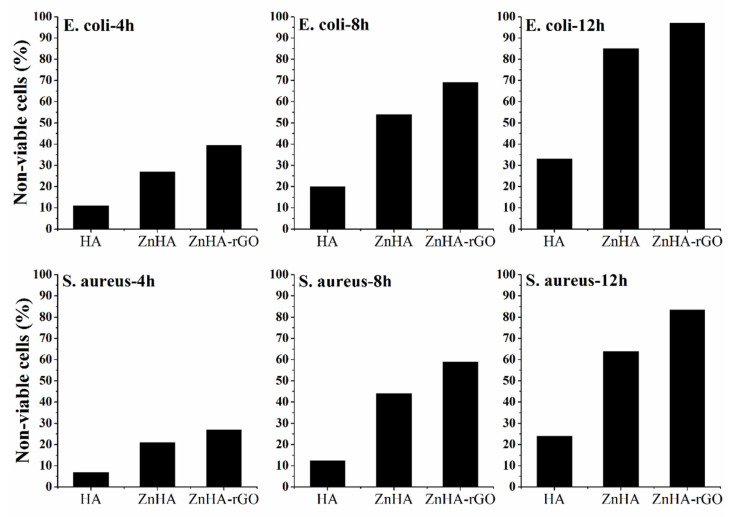
The loss of viability of *E. coli* and *S. aureus* bacteria after incubation with 100 µg mL^−1^ concentration of HA, ZnHA, and ZnHA-rGO nanoparticles for different time exposure (4, 8, and 12 h).

Figure 6 shows the time-dependent cell viability for *E. coli* and *S. aureus* bacteria for HA, ZnHA, and ZnHA-rGO nanoparticles. The loss of viability of bacterial cells was assessed by the colony counting method after every 4 h interval. As shown in Figure 6, doping Zn in HA nanoparticles significantly affected the bacterial cell loss, and the inclusion of rGO increased it further.

**Figure 7:**
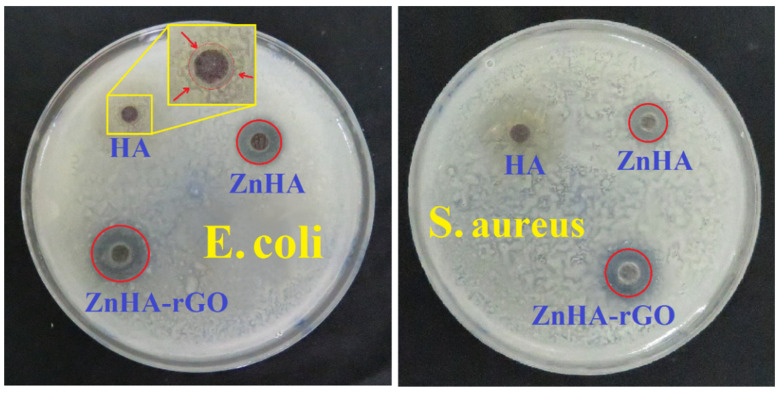
Disk diffusion tests for the evaluation of antibacterial activity of HA, ZnHA, and ZnHA-rGO nanoparticles against the *E. coli* and *S. aureus* strains. The zone of inhibition is highlighted with a red circle indicating a noticeable antibacterial effect.

Figure 7 shows the disk diffusion antibacterial test results for all three nanoparticles. The inhibition zone indicates the resistance of nanoparticles against bacterial strains. The HA sample had a small inhibition zone against *E. coli* and *S. aureus*. Doping Zn in HA and compositing it with rGO showed significant impacts on the inhibition zone. Zn in HA effectively inhibits the growth of *E. coli* and *S. aureus* on agars. The bactericidal activity of ZnHA nanoparticles can be due to the release of Zn ions, acting as an anti-bactericidal agent. Moreover, rGO further enhanced the antibacterial activity of ZnHA; a larger inhibition zone against bacteria. At the same time, the Gram-positive *S. aureus* was more resistant than Gram-negative *E. coli* for all samples.

## 3. Discussion

### 3.1. Cellular Behavior

Calcium ions in HA positively affect MSC proliferation in culture media [44]. The doped Zn in HA further promoted this phenomenon by stimulating signaling pathways that lead to boosting cell proliferation [18,19]. Simultaneous Zn doping and rGO compositing improved the adsorption of molecules on the surface of nanoparticles. It has been reported that rGO facilitates the adsorption of ions and proteins at the surface of nanoparticles [29,30], leading to improvement in cell proliferation [44].

The ALP enzyme participates in the process of maturation of osteoblast cells and alkalizes the environment, which results in hydrolyzing the phosphomonoesters to inorganic phosphates [45]. Mineralization of the organic phosphates results in the formation of ECM for the bone cells [46]. The presence of calcium, Zn ions, and rGO nanoparticles in the cell culture media can boost the activity of ALP enzymes, and as such, the differentiation of MSCs into osteoblasts would intensify [18,30].

#### 3.1.1. Effect of Hydroxyapatite on Cellular Behavior

HA can differentiate and proliferate HMSCs by stimulating intracellular signaling. Stimulation of HMSCs by HA raises the activity of the CREB transcription factor, which regulates several osteoblast marker genes. HA nanoparticles can enter the cytoplasm through the endosome and decompose into its ingredients, calcium, and phosphate [47]. Xu et al. [47] revealed that increasing the intracellular concentration of calcium ions stimulates several signaling pathways, including calcium, cAMP, Ras, Rap1, and MAPK. As a product of adenylate cyclase activity, cAMP is a secondary messenger that arouses Epac and subsequently Rap1 [48]. Rap1 has the potential to activate Raf1, and Raf1 induces phosphorylation of MEK and ERK1/2, and Raf1 also activates PI3K and then AKT, which eventually increases the CREB transcription factor [49]. Phosphorylated CREB can help mRNA expression associated with osteoblastic differentiation, such as Runx2, ALP, OCN, Col1, and OSX [50].

#### 3.1.2. Effect of Zinc on Cellular Behavior

Zn has been known as a stimulant factor of osteoblastic differentiation and proliferation in vitro and in vivo [51]. Several mechanisms have been identified for Zn-related osteogenesis, including several intracellular signaling pathways that eventually lead to the expression of genes that contribute to differentiation, mineralization, and proliferation. The mRNA and protein levels of the transcription factor Runx2, TGF-β, smad2, smad3, and BMP2 increase upon initial contact of Zn ions with HMSCs [19]. TGF-β phosphorylates smad2 and smad3 proteins by binding to TGF-β receptors. These proteins, together with smad4, lead to the proliferation of chemotaxis and early differentiation [52]. BMP2 also phosphorylates smad1, smad5, and smad8 proteins via binding to BMP2 receptors [53]. When these proteins enter the cell nucleus, genes such as DLX5, OPN, OSX, and Runx2 are transcribed for cell differentiation and proliferation [52]. On the other hand, Zn increases phosphorylated CREB through stimulation of the cAMP-PKA-CREB signaling pathway, which leads to the expression of other osteogenesis markers such as OCN, ALP, Col1, and Runx2 [54].

#### 3.1.3. Effect of rGO on Cellular Behavior

**Figure 8:**
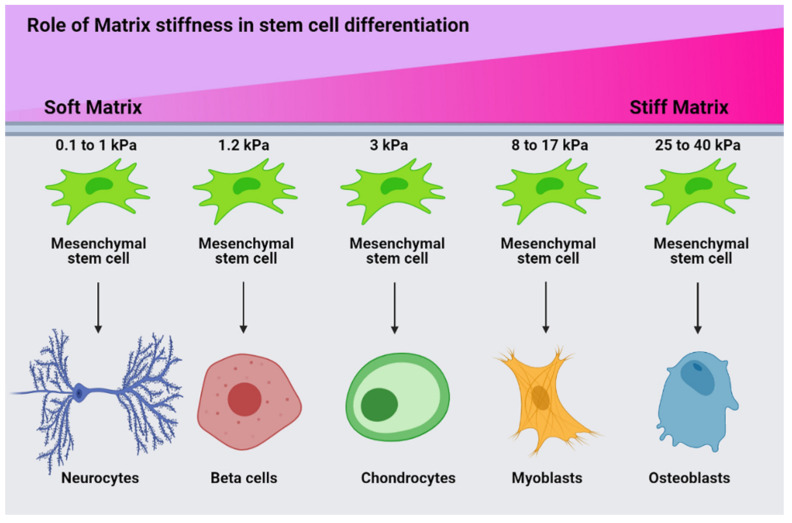
Schematic representation of the relationship between matrix stiffness and HMSC differentiation. Matrix stiffness significantly affects the differentiation of HMSCs; higher stiffness leads to osteogenic differentiation.

rGO in the ZnHA-rGO nanosystem, via interacting with the surface of HMSCs, will create optimal conditions for focal adhesion to differentiate. In addition, rGO increases bone scaffold strength and cell adhesion. The addition of graphene and its derivatives to bone scaffolds increases their mechanical strength and modulus [55]. Matrix stiffness plays a crucial role in the orientation of cell differentiation by stimulating the expression of specific genes. Hence, with increasing matrix stiffness, the expression of osteogenesis genes in HMSCs increases. The relationship between matrix stiffness and HMSC differentiation can be observed in Figure 8. Accordingly, the higher the matrix stiffness, the higher the expression of osteogenesis genes [56]. rGO in the ZnHA-rGO nanosystem increases the stiffness of ECM and helps osteoblastic differentiation of HMSCs. rGO causes actin polymerization by stimulating integrin (a protein that mediates between ECM and cytoskeleton). Integrin stimulation creates a chain of connections between the Talin, Vinculin, Zyxin, Actinin, Paxillin, Parvin, and FAK proteins. FAK helps to activate RhoA and subsequently ROCK by phosphorylating RhoGEF [57]. ROCK phosphorylates MLC, which induces actin polymerization. The actin polymerization, followed by forming actin filaments attached to myosin, causes internal forces in the cytoskeleton [58] (between the nucleus and the membrane). These internal forces lead to more calcium ions entering the cell and mechanotransduction signaling events, which activate the transcription factors YAP and TAZ [59]. Besides, FAK activates the β-catenin protein by activating the PI3K-AKT pathway, which is a major transcription factor in the expression of genes involved in osteoblast proliferation [60]. Alternatively, rGO phosphorylates p130Cas by stimulating integrin and FAK, leading to Crk activation. Finally, Crk activates JNK by stimulating Dock180 and ELMO and subsequently Rac. As a result, more activity of FOXO1 can be seen, an important transcription factor in osteoblast differentiation [61]. On the other hand, Crk activity can activate GRF2, Rap1, and Raf1, and subsequently can phosphorylate MEK and ERK, and this pathway increases phosphorylated CREB [62].

#### 3.1.4. Synergistic Effect in ZnHA-rGO Nanosystem

**Figure 9:**
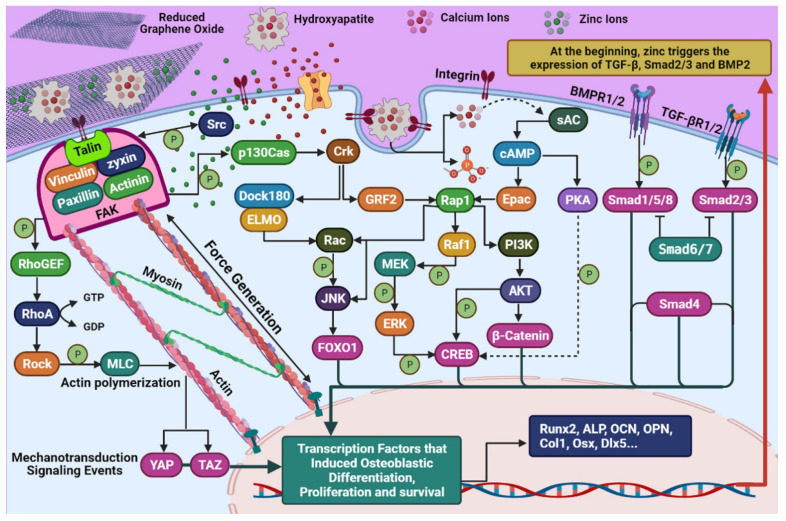
Schematic representation of ZnHA-rGO nanosystem interactions with MSC. The illustration shows the molecular mechanism of proliferation modulation and osteogenic differentiation of MSC by the ZnHA-rGO nanosystem through signaling pathways.

As a result, not only Zn and HA increase CREB-activated transcription factor levels, but also rGO has a similar effect on upregulating CREB-related genes. Generally, Zn, HA, and rGO in this nanosystem can increase the level of transcription factors related to osteogenesis by stimulating specific signaling pathways. Since each of the HA, Zn, and rGO agents individually have positive effects on osteogenesis and cell proliferation, using a combination of these agents in a nanocomposite form helps to generate a synergistic effect on the proliferation and osteogenic differentiation of HMSCs. The synergistic effect of this nanosystem leads to the activation of important transcription factors for the expression of genes related to osteogeneses such as Smad1, Smad2, Smad3, Smad4, Smad5, Smad8, β-catenin, CREB, FOXO1, TAZ, and YAP. The synergistic influence of this nanosystem on the proliferation and osteoblastic differentiation of HMSC can be observed in the schematic presented in Figure 9.

### 3.2. Antibacterial Behavior

#### 3.2.1. Effect of Hydroxyapatite on Antibacterial Behavior

The HA nanoparticles can have an antibacterial effect against both Gram-positive and Gram-negative bacteria. HA causes mechanical damage to the bacterial membrane due to its abrasive surface [63]. Electrostatic interaction between HA nanoparticles and the bacterial cell wall can lead to the entry of nanoparticles into the cytoplasm. With the entry of HA and its reformation inside the bacterium, it will cause the bacterium’s death. The antibacterial efficiency of HA nanoparticles will be increased by activation of groveling and spores [63,64]. In addition, HA increases oxidative stress by affecting the amount of ROS inside and outside the cell. Oxidative stress attacks many of the cell components inside a bacterium that are needed for life [65]. Gram-positive bacteria are also less sensitive to HA nanoparticles than Gram-negative bacteria, due to differences in membrane structure.

#### 3.2.2. Effect of Zinc on Antibacterial Behavior

The ZnHA nanoparticles attack bacteria by different mechanisms. These mechanisms are divided into two categories: direct interaction of nanoparticles with bacteria and interaction of released Zn^2+^ ions with the bacterial wall and its constituents. ZnHA nanoparticles can damage the wall and membrane of bacteria and enter the bacteria through channels, damaged areas, and accumulate in the cytoplasm. Accumulation of nanoparticles within bacteria is one of the possible mechanisms in the death of bacteria by ZnHA nanoparticles [66]. However, as shown in the antibacterial test results presented in Figure 6 and Figure 7, it has been found that Gram-positive bacteria are less sensitive to ZnHA nanoparticles than Gram-negative bacteria. The reason for this can be attributed to the greater thickness of the peptidoglycan layer in Gram-positive bacteria [67,68] in such a way that the predominant mechanism of inhibiting Gram-positive *S. aureus* bacteria by ZnHA nanoparticles can be attributed to the induction of downregulating of amino acid synthesis and dysfunction of vital bacterial enzymes [69]. Another mechanism of bacterial inhibition is related to Zn^2+^ ions released from the ZnHA-rGO nanosystem into bacteria. For example, Zn^2+^ ions can interfere with mitochondrial function or damage DNA and RNA. In addition, high concentrations of Zn^2+^ ions in bacteria may alter the three-dimensional conformation of proteins and disrupt the function of enzymes and electron transport chains [70].

#### 3.2.3. Effect of rGO on Antibacterial Behavior

**Figure 10:**
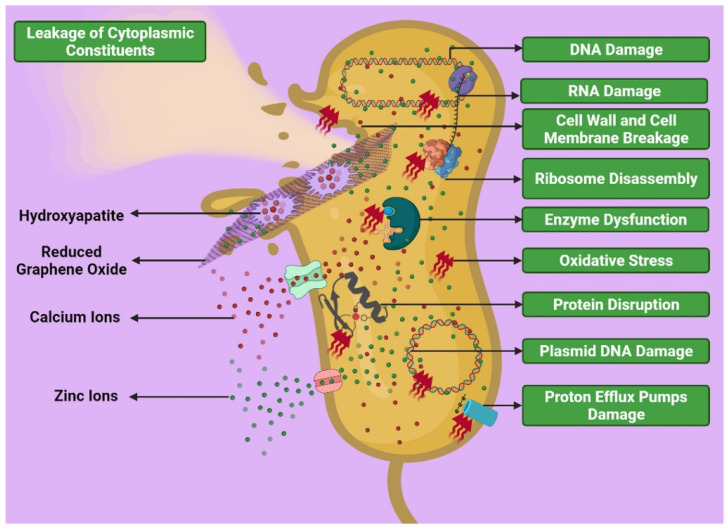
The possible interaction of the ZnHA-rGO nanosystem in the face of bacteria. The three components of the nanocomposite (HA, Zn^2+^, and rGO) damage the cell wall and intracellular components of bacteria.

rGO and GO both have antibacterial properties. rGO, as a component of the nanocomposite, has several mechanisms to inhibit bacterial growth. The principal antibacterial mechanism of rGO for Gram-negative and -positive bacteria is cell wall mechanical damage and inhibition of cell division, respectively [71]. Antibacterial mechanisms of rGO include cell entrapment, bacterial aggregation by rGO sheet, a sharp edge-mediated cutting effect that leads to rupture of the bacterial cell wall, and oxidative stress by increasing ROS levels leading to structural damage to nucleic acids and proteins [72]. Since the three components of the nanocomposite can damage the cell wall and intracellular components of bacteria, the synergistic effect of these materials creates a higher antibacterial potential for this nanosystem. The synergistic effect of the nanosystem compounds in the face of bacteria can be seen in a schematic presentation in Figure 10.

## 4. Materials and Methods

### 4.1. Material Preparations

Extraction of natural HA nanoparticles was performed according to the method presented in a previous study [34]. Doping Zn in HA nanoparticles and compositing it with rGO has also been described in a previous study [34].

### 4.2. Characterization

To study the morphology and structure of nanoparticles, TEM, XRD, and Raman spectroscopy analyses were used. Morphology of the nanopowders was analyzed using transmission electron microscopy (TEM, JEOL JEM-ARM200CFEG UHR-TEM). The XRD examination was also conducted using a single-beam, Cu Kα, λ = 1.5406 Å (PANalytical, Pro MPD model, Almelo, Netherlands). The step size and step time of XRD were considered as 0.02° and 2 s, respectively. The Raman spectra were achieved by an inVia Reflex Renishaw (Wotton-under-Edge, Gloucestershire, United Kingdom) Raman spectrometer equipped with a laser diode (λ = 785 nm, 30 mW).

### 4.3. Cellular Behavior

#### 4.3.1. Cell Culture

The initial density of 10^5^ MSCs/well was seeded in a culture plate containing Dulbecco’s modified Eagle’s medium (DMEM). Then, 10% fetal bovine serum (FBS) and 1% penicillin-streptomycin were added to the mixture. The culture media were incubated at 37 °C in a humidified atmosphere of 5% CO_2_. After overnight incubation, the cells were treated with 100 µg mL^−1^ concentrations of nanoparticles. The media were replaced with fresh ones at intervals of two days.

#### 4.3.2. Cell Viability Assessment

The proliferation of MSCs for culture media treated with nanoparticles was investigated for 7 and 14 days by MTT assay.

The MTT assay (Sigma Inc., Marlborough, MA, USA) was carried out to assess the expansion of MSCs cultured on 24 well plates. At the first step, 5 mg of MTT powder was dissolved in phosphate-buffered saline (PBS) solution. To dilute the MTT solution in the second step, 900 μL of DMEM/F12 medium was added to the solution. In the third step, 1 mL of the prepared solution was poured into each well to form the Formazan crystals and kept in dark incubation for 2 h. Then, the supernatants of the wells were removed completely, and for dissolving the prepared crystals, 1 mL of a stabilized solution (including 10% Triton x-100, 0.1 M HCl, and isopropanol) was added to each well. Finally, a microplate (Elx808, BioTek, Winooski, VT, USA) at a wavelength of 570 nm was used to read the absorbance of each well sample.

#### 4.3.3. Alkaline Phosphatase Activity

ALP is an osteogenic marker whose activation is intensified in the bone growth duration; the expression value of ALP rises during the beginning of differentiation of MSCs [45,46]. Such an increment of ALP expression develops bone formation by nucleating calcium phosphate on ECM, causing mineralization, and acting as an essential function in osteoblast differentiation and maturation [45,46].

The HA, ZnHA, and ZnHA-rGO nanoparticles were exposed to MSCs for 7 and 14 days to assess their ALP activities. After incubation, the MSCs were washed with PBS, lysed by lysis buffer, incubated for 30 min at 37 °C, and held for 12 h at 4 °C. Afterwards, the cells were centrifuged for 10 min at 12,000 rpm. Then, the supernatant and p-nitrophenyl phosphate solution (1 to 20) were mixed and incubated for 1 h at 25 °C. The ALP activity was determined utilizing the fluorescence microscope system (Cytation 5, BioTek, Winooski, VT, USA) at a wavelength of 405 nm.

### 4.4. Antibacterial Activity

To assess antibacterial activities, the samples were tested using both *E. coli* and *S. aureus* bacteria, because these are the two most common bacteria in any hospital environment that can cause bacterial infection. Moreover, bacteria belonging to both groups were selected; Gram-positive (*E. coli*) and Gram-negative (*S. aureus*).

#### 4.4.1. Cell Viability Assessment

The nanoparticles were dispersed in deionized (DI) water with concentration of 100 µg mL^−1^. In addition, dilution 10^6^ CFU/mL of bacteria were incubated with dispersed nanoparticles in DI water at 37 °C under 150 rpm stirring speed for up to 6 h. Bacterial samples in DI water without nanoparticles were considered as the control. Eventually, 100 µL of each sample was spread onto LB plates after each 4 h of time interval and left to grow for 12 h at 37 °C. Colonies were counted and compared with those of the control samples to determine the total viable count and percentage of the non-viable cells.

#### 4.4.2. Disk Diffusion Method

The disk diffusion test was carried out by pouring lysogeny broth and brain heart infusion agar into Petri dishes. The nanoparticles (50 mg) suspended in ethanol were applied to sterilized filter paper disks (6 mm in diameter). Bacteria suspension (100 µL) was spread uniformly on the surface of solidified nutrient agar, followed by placing sterilized disk specimens onto the agar dishes. Finally, samples were placed in an incubator with a humidified atmosphere of 5% CO_2_ and 95% air for 18 h at 37 °C. All disk diffusion tests were performed in triplicate, and the diameters (mm) of the inhibition zone were measured after incubation.

## 5. Conclusions

In this work, HA nanoparticles extracted from bovine cortical bone were doped with Zn by mechanical alloying. The ZnHA nanoparticles were also composited with rGO by photochemical reduction. The microstructural studies confirmed the hexagonal crystal structure for HA and ZnHA nanoparticles. The influence of HA nanoparticles on stem cell behavior revealed no toxic effect, and significantly improved cell proliferation and alkaline phosphatase activity, and that Zn and rGO have a synergistic effect on these behaviors. The antibacterial test results showed that HA nanoparticles have antibacterial function against Gram-negative (E. coli) and Gram-positive (*S. aureus*) bacteria. The antibacterial activity of HA nanoparticles was significantly increased by Zn doping such that the bacterial cell loss raised to 3-fold in 12 h. Finally, rGO also had a positive effect on increasing the antibacterial activity of the ZnHA nanoparticles.

## Data Availability

All data generated or analyzed during this study are included in the present article.
